# The Effects of Prebiotic Supplementation with OMNi-LOGiC^®^ FIBRE on Fecal Microbiome, Fecal Volatile Organic Compounds, and Gut Permeability in Murine Neuroblastoma-Induced Tumor-Associated Cachexia

**DOI:** 10.3390/nu12072029

**Published:** 2020-07-08

**Authors:** Beate Obermüller, Georg Singer, Bernhard Kienesberger, Ingeborg Klymiuk, Daniela Sperl, Vanessa Stadlbauer, Angela Horvath, Wolfram Miekisch, Peter Gierschner, Reingard Grabherr, Hans-Jürgen Gruber, Maria D. Semeraro, Holger Till, Christoph Castellani

**Affiliations:** 1 Department of Paediatric and Adolescent Surgery, Medical University of Graz, 8036 Graz, Austria; beate.obermueller@medunigraz.at (B.O.); bernhard.kienesberger@medunigraz.at (B.K.); holger.till@medunigraz.at (H.T.); christoph.castellani@medunigraz.at (C.C.); 2 Department of Biomedical Research, Medical University of Graz, 8036 Graz, Austria; 3 Core Facility Molecular Biology, Center for Medical Research, Medical University of Graz, 8036 Graz, Austria; ingeborg.klymiuk@medunigraz.at; 4 Division of Paediatric Haematology and Oncology, Department of Pediatrics and Adolescent Medicine, Medical University of Graz, 8036 Graz, Austria; daniela.sperl@medunigraz.at; 5 Division of Gastroenterology and Hepatology, Department of Internal Medicine, Medical University of Graz, 8036 Graz, Austria; vanessa.stadlbauer@medunigraz.at (V.S.); angela.horvath@medunigraz.at (A.H.); 6 Center for Biomarker Research in Medicine (CBmed), 8036 Graz, Austria; 7 Rostock Medical Breath Research Analytics and Technologies (ROMBAT), Department of Anesthesiology and Intensive Care, Rostock University Medical Center, 18057 Rostock, Germany; wolfram.miekisch@uni-rostock.de (W.M.); peter.gierschner@gmx.net (P.G.); 8 Department of Biotechnology, University of Natural Resources and Life Sciences, 1190 Vienna, Austria; reingard.grabherr@boku.ac.at; 9 Clinical Institute of Medical and Chemical Laboratory Diagnostics, Medical University of Graz, 8036 Graz, Austria; hans.gruber@medunigraz.at (H.-J.G.); maria.semeraro@medunigraz.at (M.D.S.)

**Keywords:** prebiotics, gut permeability, neuroblastoma, microbiome, volatile organic compounds

## Abstract

Malignant diseases can cause tumor-associated cachexia (TAC). Supplementation with prebiotic non-digestible carbohydrates exerts positive metabolic effects in experimental oncologic diseases. The aim of this project was to assess the effect of prebiotic supplementation with OMNi-LOGiC^®^ FIBRE on intestinal microbiome, bacterial metabolism, gut permeability, and inflammation in a murine model of neuroblastoma (NB)-associated TAC. For this study, 2,000,000 NB cells (MHH-NB11) were implanted into athymic mice followed by daily supplementation with water or 200 mg prebiotic oligosaccharide (POS) OMNi-LOGiC^®^ FIBRE (NB-Aqua, *n* = 12; NB-POS, *n* = 12). Three animals of each tumor group did not develop NB. The median time of tumor growth (first visibility to euthanasia) was 37 days (IQR 12.5 days) in the NB-Aqua group and 37 days (IQR 36.5 days) in the NB-POS group (*p* = 0.791). At euthanasia, fecal microbiome and volatile organic compounds (VOCs), gut permeability (fluorescein isothiocyanate-dextran (FITC-dextran), and gut barrier markers were measured. Values were compared to sham animals following injection of culture medium and gavage of either water or OMNi-LOGiC^®^ FIBRE (SH-Aqua, *n* = 10; SH-POS, *n* = 10). Alpha diversity did not differ significantly between the groups. Principal coordinate analysis (PCoA) revealed clustering differences between Aqua and POS animals. Both NB and POS supplementation led to taxonomic alterations of the fecal microbiome. Of 49 VOCs, 22 showed significant differences between the groups. NB animals had significantly higher gut permeability than Aqua animals; POS did not ameliorate these changes. The pore and leak pathways of tight junctions did not differ between groups. In conclusion, our results suggest that NB-induced TAC causes increased gut permeability coupled with compositional changes in the fecal microbiome and VOC profile. Prebiotic supplementation with OMNi-LOGiC^®^ FIBRE seemed to induce modifications of the fecal microbiome and VOC profile but did not improve gut permeability.

## 1. Introduction

The metabolic competition for nutrients between host and tumor has increasingly moved into the focus of science in the past years. In end-stage malignant diseases, this may lead to tumor-associated cachexia (TAC) characterized by wasting of adipose and muscle tissue [1]. Depending on the underlying type of cancer, TAC affects up to three-quarters of oncologic patients [2]. Among others, TAC is associated with an increased risk of complications as well as higher hospitalization rates and mortality (reviewed in [3]). The causes of TAC are complex and the underlying molecular mechanisms have still not been elucidated in detail [3]. However, an interaction between host and tumor involving the secretion of pro-inflammatory cytokines from both cancer cells and the host immune system has been shown to be involved. These soluble mediators include tumor necrosis factor alpha (TNF-α), interferon gamma (INF-γ), and a variety of interleukins (IL-6, IL-8, IL-1β, and others) [4,5,6]. Additionally, the tumor itself may release catabolic substances such as proteolysis-inducing factor, anemia-inducing substance, activin, or myostatin, thereby further fueling the progression of TAC [4,7]. 

Although the tumor itself seems to play the major role in development and progression of TAC, the intestinal microbiome and its interaction with gut barrier, inflammation, and metabolism have also been implicated in the pathogenesis of TAC [5,8,9,10,11,12]. Among others, the intestinal microbiome serves as source for bacterial metabolites such as short-chain fatty acids and their derivatives, is involved in bile acid metabolism, and secretes microbe-associated molecular patterns (MAMPs) [13,14,15]. In oncologic diseases, these substances may influence the patients’ nutrient uptake, metabolism, gut motility, gut barrier function, and systemic inflammatory responses in general [13]. Alterations of the intestinal microbiome, especially decreased levels of *Lactobacillus*, have already been demonstrated in animal models of TAC [8,11]. The gut barrier function may represent a possible link between the intestinal microbiome and the inflammatory response. The pro-inflammatory state associated with TAC [16] but also alterations of the composition of the intestinal microbiome or the bacterial metabolism [17,18] may cause disturbances of intestinal epithelial cells (IECs), resulting in increased gut permeability. Consequently, an increased gut permeability based on intestinal cell apoptosis has already been shown in a murine model of TAC [11]. The increased permeability may cause passage of MAMPs like lipopolysaccharide into the blood stream, causing further aggravation of inflammation and sarcopenia through activation of toll-like receptors or nucleotide-binding oligomerization domain-containing proteins 1 and 2 [13,16,19,20].

Unfortunately, the TAC-associated loss of body weight cannot be reversed by nutritional support alone, resulting in progressive functional impairment [3]. Thus, recent research has focused on possibilities to interrupt the vicious cycle of catabolism and to attenuate the effects of TAC. In experimental animal models, modification of the intestinal microbiome using either pre- or probiotics seems to have beneficial effects on the host [8,9,10]. Positive metabolic effects of treatment with non-digestible carbohydrates with prebiotic properties have already been demonstrated. Pectin, for instance, seems to reduce anorexia and lipolysis and cancer cell invasion in the liver in a BaF3 leukemia model in mice [9]. However, the effects of prebiotics on gut barrier function and bacterial metabolites in the setting of TAC have not yet been examined.

The aim of this project was therefore to assess the effect of prebiotic supplementation with OMNi-LOGiC^®^ FIBRE on intestinal microbiome, bacterial metabolism, gut permeability, and inflammation in a rodent model of neuroblastoma (NB)-associated TAC.

## 2. Materials and Methods

For this study, 44 BALB/c Rj:ATHYM-Foxn1nu/nu male mice were obtained from Janvier Labs (Le Genest-Saint-Isle, France) at an age of 7 weeks. Mice were kept single in individually ventilated cages under specific pathogen-free conditions according to the FELASA guidelines 2014. Animals had free access to food and water at all times and were subjected to a 12 h light and dark cycle. Animals were allowed to adapt to local conditions for 10 days prior to further interventions. All animal experiments were approved by the veterinary board of the Austrian Federal Ministry of Education, Science and Research (BMBWF 66.010/0127-V-3b/2018). 

### 2.1. Cell Culture

Human NB cells (cell line MHH-NB11) were obtained from DSMZ laboratories (Leibnitz Institute DSMZ – German Collection of Microorganisms and Cell Cultures, Braunschweig, Germany; Cat. No. ACC157,lot no. 5). Cells were cultured in RPMI medium (RPMI-1640 Medium, Sigma-Aldrich Handels GmbH, Vienna, Austria) with 2 mM glutamine supplemented with 10% fetal bovine serum (Sigma-Aldrich Handels GmbH, Vienna, Austria) and 1X minimum essential medium eagle (Sigma-Aldrich Handels GmbH, Vienna, Austria) nonessential amino acids as described previously [5]. After six passages, cell entity was verified by STR analysis with a Promega PowerPlex 16HS System (Promega GmbH, Braunschweig, Germany; Cat. No. DC2101) and mycoplasma infection was ruled out by PCR using a commercially available kit (Venor GeM Mycoplasma Detection Kit, Bioproducts, Stockerau, Austria). Thereafter, cells were diluted to a concentration of 500,000 cells per 0.1 ml culture medium.

### 2.2. Tumor Cell Inoculation and Tumor Growth

At an age of 8 weeks, mice were allocated to sham and tumor groups aiming for equal body weight distributions in the respective groups. Animals of the sham group (SH animals) received 4 subcutaneous depots of 0.1 ml cell culture medium per depot on the back using a 25G needle. Animals of the tumor group (NB animals) were injected with 4 depots of tumor cells with 0.1 ml (equaling 500,000 cells) per depot. 

After injection, animals received daily gavage of 300 µL autoclaved water (SH-Aqua, *n* = 10; NB-Aqua, *n* = 12) or of 200 mg OMNi-LOGiC^®^ FIBRE (prebiotic oligosaccharide (POS), containing 5 g dextrin and 5 g guar gum per 10 g powder; kindly provided by Institut AllergoSan, Graz, Austria) dissolved in 300 µL autoclaved water (SH-POS, *n* = 10; NB-POS, *n* = 12). 

Tumors were allowed to grow until mice showed clinical signs of cachexia including loss of adipose tissue, impaired mobility, and impaired ability to access food due to tumor size. In this case, mice were scheduled for euthanasia. 

### 2.3. Euthanasia and Sample Collection

Sham mice were euthanized in equal numbers at the same time points. Sixteen hours prior to euthanasia, stool samples were obtained and stored at −80 °C for subsequent microbiome analysis. Thereafter, mice were gavage fed with 500 mg/kg fluorescein isothiocyanate-dextran 4kD (FITC-Dextran 4SD, Sigma-Aldrich Handels GesmbH, Vienna, Austria) dissolved in PBS buffer at a concentration of 50 mg/mL. Stool samples were obtained from all mice at the same time on the day of euthanasia, weighed, and stored in standard glass vials (Gerstel GmbH, Muelheim an der Ruhr, Germany) at 6 °C for subsequent volatile organic compound (VOC) analysis. At the same time, ambient room air samples were collected (vials were unscrewed and left open for 20 min in the same room) and stored under the same conditions. Under sedation with 5% inhaled isoflurane (Forane^®^, Baxter Healthcare GmbH, Vienna, Austria), mice underwent exsanguinating blood sampling by cardiac puncture. Blood was drawn to standard serum vials and allowed to clot for 30 min. Then, blood samples were centrifuged at 6 °C at 10,000 rpm for 10 min. Supernatant serum was harvested and aliquoted to standard Eppendorf vials. Mice were sacrificed by cranio-cervical dislocation.

The gonadal, peri-renal, and inguinal white adipose tissue (WAT) were dissected, combined, and weighed as previously described [5]. The following organs were excised for further analysis: liver, spleen, kidney, triceps surae muscle, right tibia, and 3 cm ileum (beginning 1 cm orally of the ileocecal valve). The tibia was stored in 0.1 N sodium hydroxide buffered with 1% sodium dodecyl sulfate at 60 °C for 12 h to remove all soft tissues. All solid organs, muscle, and white adipose tissue were weighed and normalized for tibial length as previously reported [11]. All specimens were labelled with the mouse number blinding examiners for the experimental groups.

### 2.4. Inflammatory Response

A commercially available Luminex^®^ magnetic bead assay (MMHMAG-70K, Merck Chemicals and Life Science GmbH, Vienna, Austria) was configured to detect interleukins (IL)-1α, -1β, and -6, macrophage inflammatory proteins (MIP)-1α, -1β, and -2, and TNF-α from serum samples. Additionally, transforming growth factors (TGF)-β1 and -β2 were assessed with an ELISA kit (TGFBMAG-64K-03, Merck Chemicals and Life Science GmbH, Vienna, Austria). All tests were performed as described in the manufacturer’s instructions.

### 2.5. Fecal Microbiome Analysis

Fecal microbiome analysis was conducted by 16S rRNA gene sequencing for the variable regions V4-V5 as published in Klymiuk et al. [21] with modifications. Briefly, total DNA was isolated using the Magna Pure LC DNA III Isolation Kit (Bacteria, Fungi) (Roche, Mannheim, Germany); mouse fecal samples were mixed with 500 µL PBS and 250 µL bacterial and mechanically lysed in Magna Lyser Green bead tubes at 6,500 rpm for 30 seconds. After enzymatic lysis with lysozyme (25 µL at 100 mg/mL) and proteinase K (43.4 µL at 20 mg/mL), samples were heat inactivated at 95 °C for 10 min and 100 µL of the suspension was isolated in the Magna Pure LC according to the manufacturer’s instructions. Total DNA was eluted in 100 µL elution buffer and stored at −20 °C until analysis. For PCR amplification, 2 µL of total DNA were used in a 25 µL PCR reaction as described in Klymiuk et al. [21] with the target specific primers 515F-GTGYCAGCMGCCGCGGTAA and 926R-CCGYCAATTYMTTTRAGTTT for 30 cycles at 55 °C annealing temperature in triplicates. Triplicates were pooled, normalized, and indexed according to the published procedure. The final 9 nM library was sequenced on an Illumina MiSeq Desktop Sequencer with 20% Phix and with version 3 chemistry for 600 cycles. FASTQ files were used for data analysis. A total of 4,130,177 paired-end raw reads were used for quality filtering and were denoised, dereplicated, merged, and checked for chimeras using the DADA2 pipeline [22] with standard settings as implemented in the QIIME2 2018.7 microbiome bioinformatics platform [23]. For taxonomic assignment, the DADA2 representative sequence set was provided with the QIIME2 classifier against SILVA rRNA database Release 132 at 99% identity [24]. 

To interpret and compare taxonomic information, 16S rRNA data were transferred to the Calypso online software (Calypso 8.84^®^, accessible through http://cgenome.net/wiki/index.php/Calypso) [25]. Samples were rarefied to a read depth of 35,193. Alpha diversity was calculated using Chao1 estimator and Shannon index. Beta diversity was examined using a redundancy analysis (RDA) and colored principal coordinate analysis (PCoA) plots based on the Bray–Curtis dissimilarity score. *p*-values were adjusted for multiple testing by false discovery rate (FDR). The identification of discriminating taxa between the groups was performed with a linear discriminant effect size (LEfSe) analysis. Differentially abundant taxa identified by LEfSe analysis were considered relevant if the differences between groups could be verified by ANOVA (*p* < 0.1).

### 2.6. Fecal Volatile Organic Compound (VOC) Analysis

For VOC analysis, specimens were sent to our laboratory partner in Rostock by overnight express maintaining a temperature of 6-8 °C and examined within 48 h after harvesting. VOC analysis was performed in the headspace of stool samples as previously reported [5,26,27]. VOCs were pre-concentrated with a commercially available solid-phase microextraction (SPME) fiber (carboxen/polymethylsiloxane, Sulpeco, Bellefonte, PA, USA). An Agilent 7890 A gas chromatograph (GC) coupled to an Agilent 5975 C inert XL mass selective detector (MSD) (Agilent, Santa Clara, CA, US) was used to separate and detect the VOCs desorbed from the SPME device. Detected marker substances were identified by means of a mass spectral library (National Institute of Standards and Technology 2005; NIST 2005, Gatesburg, PA, USA). The responses of a selected m/q ratio at a defined retention time for each substance were recorded, peak areas integrated, corrected for the weight of the fecal sample, and used for group comparison. External standards and quality controls were used in each of the measurement series to confirm the results of the VOC measurements.

### 2.7. Gut Permeability Assay

For FITC analysis, serum aliquots were protected from light and stored at 6 °C until measurement. FITC-dextran serum levels were determined photometrically within 8 h after harvesting (FLUOstar Omega, BMG LABTECH, Ortenberg, Germany) at an extinction of 485 and 535 nm. Standard curves were obtained according to the manufacturer’s protocol.

### 2.8. Investigation of Gut Barrier Markers

Total RNA from frozen mouse ileum segments was isolated with the Qiagen miRNeasy Micro Kit (Qiagen, Hilden, Germany) by DNAse treatment on column (Qiagen, Hilden, Germany) according to the manufacturer’s instructions. RNA yield was quantified on a NanoDrop 2000c Spectrophotometer (ThermoFisher Scientific, Waltham, MA, USA). For reverse transcription, 2 µg of total RNA were used in the High Capacity cDNA Reverse Transcription Kit (ThermoFisher Scientific, Waltham, MA, USA) according to the manufacturer’s instructions. The cDNA product was used as template for quantitative RT-PCR reactions in a BioRad CFX 384 real-time PCR detection system with the assays *β-actin* (*Actb*; Mm00607939_s1), *hydroxymethylbilane* (*Hmbs*; Mm01143545_m1), *tight junction protein 1* (*Tjp1*; Mm00493699_m1), *occludin-1* (*Ocln1*; Mm00500912_m1), *claudin 4* (*Cldn4*; Mm00515514_s1), *claudin 2* (*Cldn2*; Mm00516703_s1), *mucin 2* (*MUC2*; Mm01276696_m1), *mucin 3* (*MUC3*; Mm01207064_m1), and *myosin light chain kinase* (*MLCK*; Mm00653039_m1) (ThermoFisher Scientific, Waltham, MA, USA). Briefly, in 10 µL reactions 4 µL cDNA were used in triplicates in a PCR reaction with 5 µL TaqMan Genexpression MasterMix (ThermoFisher Scientific, Waltham, MA, USA), 0.5 µL assay, and 0.5 µL dH_2_O. Cycling conditions were of initial UDG incubation at 50 °C for 2 min, enzyme activation at 95 °C for 10 seconds, followed by 40 cycles of denaturation at 95 °C for 15 seconds and annealing and extension at 60 °C for one minute. *β-actin* and *Hmbs* genes were used for normalization. 

### 2.9. Bowel Wall Inflammation and Intestinal Cell Apoptosis

For visual determination of bowel wall inflammation, ileum sections underwent standard histological processing and hematoxylin and eosin staining. The morphology and inflammation in the bowel wall were classified using the Marsh–Oberhuber score (MOS) as described in the literature [28]. TUNEL staining (ab206386, abcam) was conducted to visually determine the amount of apoptotic IECs in the crypts of the ileum samples. For this evaluation, two representative regions were selected for each specimen and two specimens per mouse were analyzed at 200× magnification. This analysis was supplemented by a PCR for apoptosis genes *Bax* (*Bax*; Mm00432051_m1), *Bad* (*Bad*; Mm00432042_m1), *caspase 3* (*Casp3*; Mm0195085_m1), *lamin B1* (*Lmnb1*; Mm0521949_m1), *Bak1* (*Bak1*; Mm00432045_m1), and *Bcl2* (*Bcl2*; Mm00477631_m1) from ileal samples performed as described above (ThermoFisher Scientific, Waltham, MA, USA). 

### 2.10. Statistical Analysis

Data was managed with Microsoft Excel 2016^®^ (Microsoft Corporation, Redmond, WA, USA) spreadsheets. For statistical analysis, data was transferred to SPSS 26.0 (IBM Corporation, Armonk, NY, USA). Metric data are displayed as median and interquartile range (IQR), ordinal data as numbers and percent. Non-parametric Kruskal–Wallis tests were applied to search for global statistical differences. In case of significant results, this was followed by pairwise comparison with a Mann–Whitney U test applying a Bonferroni correction for multiple testing. Spearman’s rho test was applied for correlation analysis. Additionally, multiple linear regression analysis was run to identify factors that are associated with increased gut permeability. Graphical workup was conducted with Prism 8.4.0^®^ (GraphPad, San Diego, CA, USA). *p*-values < 0.05 were considered statistically significant.

## 3. Results

**Figure 1:**
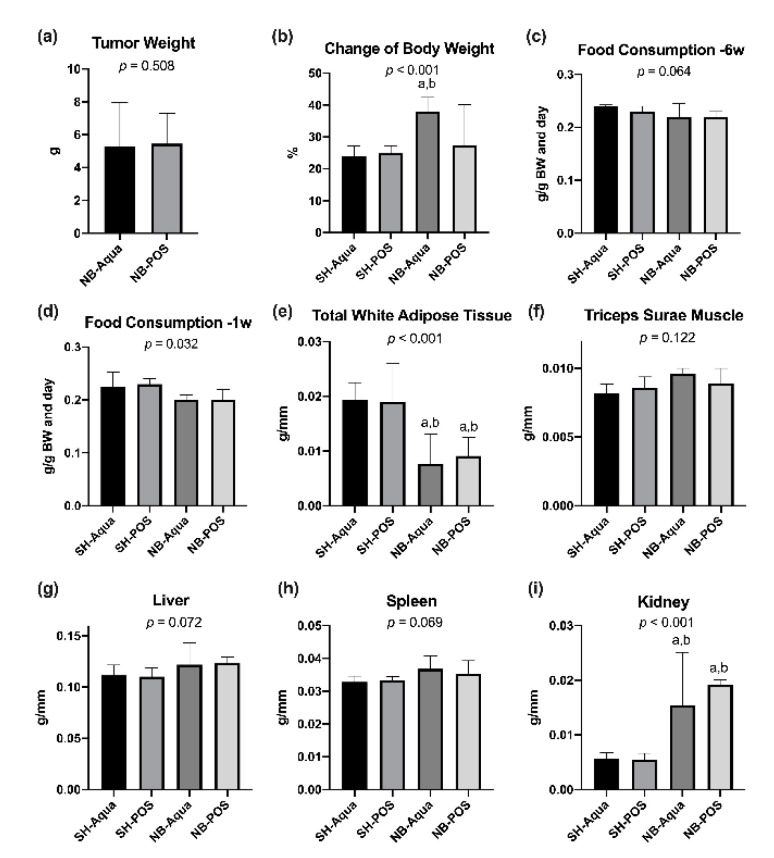
(**a**) Comparison of tumor weight; changes in (**b**) body weight, food consumption (**c**) 6 weeks and (**d**) one week prior to euthanasia; normalized weight of (**e**) total white adipose tissue, (**f**) triceps surae muscle, (**g**) liver, (**h**) spleen, and (**i**) kidney. NB—neuroblastoma; SH—sham; POS—prebiotic oligosaccharide; a—*p* < 0.05 vs. SH-Aqua; b—*p* < 0.05 vs. SH-POS.

Three animals of the NB-Aqua group and three of the NB-POS group did not develop tumors, leaving nine animals for further analysis in each of these groups. Median time to tumor onset (first visibility of tumor) was 56 days (IQR 51) in NB-Aqua and 59 days (IQR 52) in NB-POS mice (*p* = 0.825). The median time of tumor growth (first visibility to euthanasia) was 37 days (IQR 12.5 days) in the NB-Aqua group and 37 days (IQR 36.5 days) in the NB-POS group (*p* = 0.791). Tumor weight did not differ significantly between the two groups (Figure 1a, *p* = 0.508). Due to the tumor load, the body weight increased to a higher extent in the tumor groups compared to the control groups within eight weeks prior to euthanasia (Figure 1b, global significance *p* < 0.001). In the pairwise test, however, only the difference between NB-Aqua versus SH-Aqua and SH-POS reached statistical significance after correction for multiple testing. Food consumption six weeks before euthanasia did not differ significantly between the groups (Figure 1c). In contrast, one week prior to euthanasia food consumption was lower in tumor animals (global significance *p* = 0.032, Figure 1d). However, due to correction for multiple testing, the pairwise comparisons yielded no statistically significant differences between the groups. Tumor animals (NB-Aqua and NB-POS) exhibited a significant lipolysis but no sarcopenia (Figure 1e,f). The weights of liver and spleen did not differ significantly between the groups. However, kidneys were significantly heavier in tumor animals compared to sham mice (Figure 1g–i).

**Figure 2:**
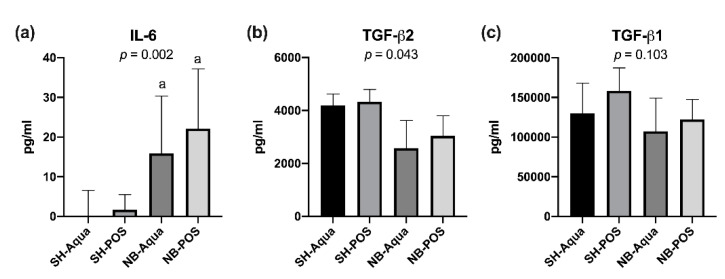
Serum levels of (**a**) IL-6, (**b**) TGF-β2, and (**c**) TGF-β1. NB—neuroblastoma; SH—sham; POS—prebiotic oligosaccharide; a—*p* < 0.05 vs. SH-Aqua.

The cachectic state of NB animals was accompanied by a significant increase of pro-inflammatory IL-6. Anti-inflammatory TGF-β2 was lower in tumor animals (global significance *p* = 0.043), but due to correction for multiple testing the pairwise comparisons yielded no statistically significant differences between the groups. TGF-β1 did not differ between the groups (Figure 2a–c). Likewise, the remaining cytokines did not show statistically significant differences. Treatment with POS did not improve the aforementioned parameters. 

### 3.1. Fecal Microbiome Analysis

**Figure 3:**
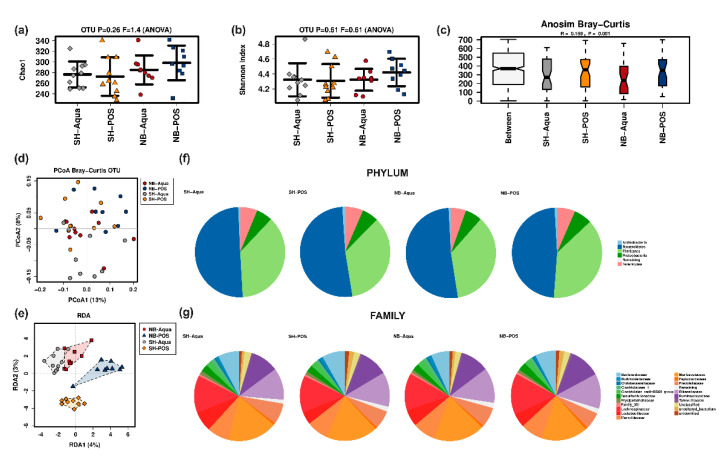
(**a**) Chao1 and (**b**) Shannon index; (**c**) Anosim Bray–Curtis, (**d**) PCoA Bray–Curtis, (**e**) redundancy analysis (RDA) and relative abundances at the (**f**) phylum and (**g**) family level. NB—neuroblastoma; SH—sham; POS—prebiotic oligosaccharide.

Alpha diversity markers (Chao1 estimator (Figure 3a) and Shannon index (Figure 3b)) were not significantly different between the groups. Beta diversity analysis showed significant differences. Bray–Curtis was significantly higher between groups than within individual groups, indicative of clustering (Anosim, R = 0.159, *p* = 0.001) (Figure 3c). PCoA analysis revealed clustering differences between Aqua and POS animals (Figure 3d). Redundancy analysis (RDA) confirmed the significant influence of TAC and POS on microbiome composition (F 1.29; Var 69.06; *p* = 0.001) (Figure 3e). Pairwise analyses of beta diversity are shown in Figure S1, Supplementary Materials. At the phylum level, NB-POS animals had a higher abundance of Firmicutes and lower abundance of Bacteroidetes compared to the other groups (Figure 3f). The comparison of the Bacteroidetes to Firmicutes ratio, however, did not reach statistical significance (*p* = 0.261). Figure 3g depicts relative abundances at the family levels showing significant global differences for Lactobacillaceae (*p* = 0.009) and Clostridiaceae (*p* = 0.025). Pairwise comparison corrected for multiple testing revealed significantly higher levels of Lactobacillaceae in SH-Aqua compared to SH-POS (*p* = 0.009) and of Clostridiaceae in SH-Aqua compared to SH-POS (*p* = 0.040).

**Figure 4:**
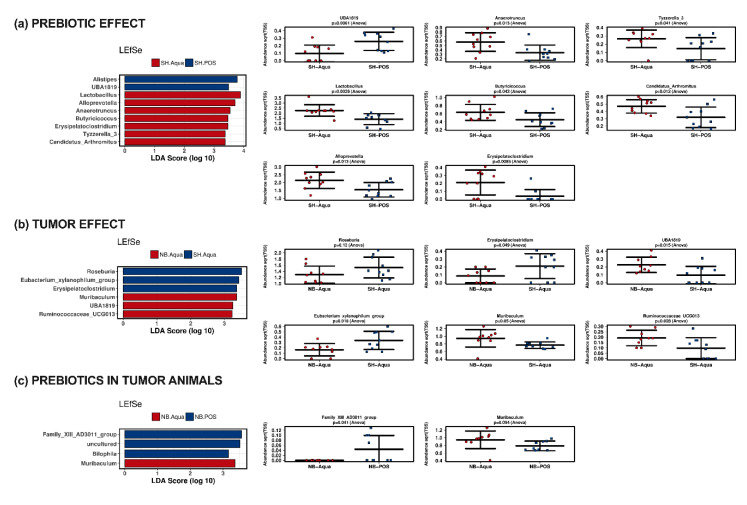
Linear discriminant effect size (LEfSe) analysis examining the discriminating effect of different bacteria regarding the effect of (**a**) prebiotic supplementation, (**b**) the tumor, and (**c**) prebiotic gavage in tumor animals. Only bacteria with differences between the groups corresponding to a *p*-value of <0.1 are displayed as strip charts. NB—neuroblastoma; SH—sham; POS—prebiotic oligosaccharide.

LEfSe analyses at the genus level were conducted to determine the effects of prebiotic treatment (SH-Aqua compared to SH-POS; Figure 4a), the effect of the tumor (SH-Aqua compared to NB-Aqua; Figure 4b), and the effect of prebiotic supplementation in tumor animals (NB-Aqua compared to NB-POS, Figure 4c). Investigating the effect of OMNi-LOGiC^®^ FIBRE, LEfSe at the genus level revealed increased abundances of *Alistipes, UBA1819* and decreased abundances of *Lactobacillus*, *Alloprevotella*, *Anaerotruncus*, *Butyricicoccus*, *Erysipelatoclostridium*, *Tyzzerella 3*, and *Candidatus arthromitus* in SH-POS. Tumor animals with TAC (NB-Aqua) could be discriminated from control animals (SH-Aqua) by decreased abundances of *Roseburia*, *Eubacterium xylanophilum* group, and *Erysipelatoclostridium* and increased abundances of *Muribaculum, UBA1819* and *Ruminococcaceae UCG013*. Tumor animals receiving gavage of prebiotics could be discriminated from those receiving water by increased abundance of *Clostridium* family AD3011 group and *Bilophila* as well as decreased abundance of *Muribaculum*.

### 3.2. Fecal VOC Analysis

**Figure 5:**
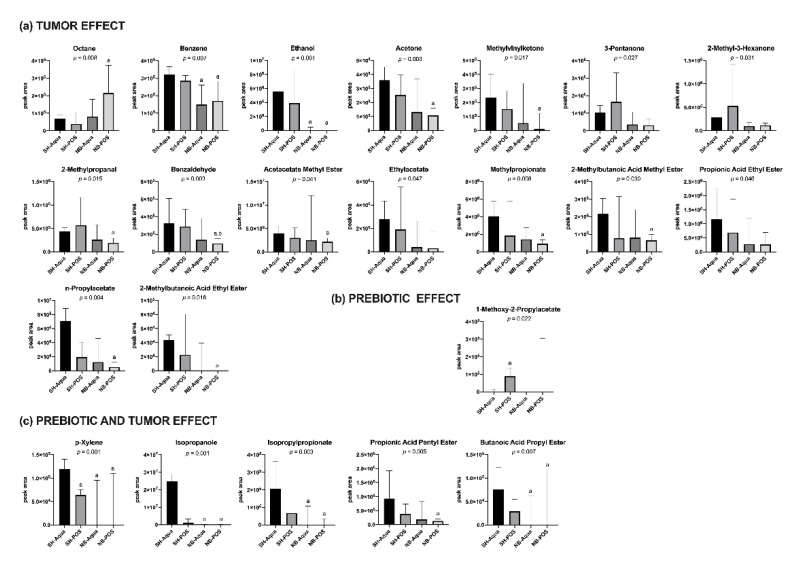
Fecal volatile organic compound (VOC) profiles of the different groups split into (**a**) tumor effect, (**b**) prebiotic effect, and (**c**) prebiotic and tumor effect. NB—neuroblastoma; SH—sham; POS—prebiotic oligosaccharide; a—*p* < 0.05 vs. SH-Aqua; b—*p* < 0.05 vs. SH-POS.

GC-MS allowed detection of a total of 53 different substances. Of these, four could be attributed to contamination by ambient room air, leaving 49 VOCs for group comparison. The majority of these substances belonged to the group of esters, but also alkanes, cyclic carbohydrates, ketones, and sulphur-containing substances were found. Of the 49 VOCs, 22 showed significant differences between the groups. While the majority of substances were associated with TAC (Figure 5a), 1-methoxy-2-propylacetate was predominantly associated with prebiotic treatment (Figure 5b). p-xylene, isopropanol, isopropylpropionate, propionic acid pentyl ester, and butanoic acid pentyl ester appeared to be influenced by both tumor and gavage of prebiotic fibers (Figure 5c). 

In the correlation analysis, ethanol levels negatively correlated with the relative abundance of *Muribaculum* and positively with *Roseburia* and *Eubacterium* (Table S1, Supplementary Materials). The alkanes octane and pentane and the aldehydes acetaldehyde and benzaldehyde positively correlated with liver weight. The cyclic compounds benzene and p-xylene, the alcohols ethanol and isopropanol, the ketones acetone, methylvinylketone, 2-pentanone, and 2-hexanone, and most of the esters negatively correlated with liver weight (Table S2, Supplementary Materials). Total WAT positively correlated with benzene, and negatively with pentane, ethanol, isopropanol, acetone, methylvinylketone, 3-pentanone, 2-pentanone, 2,3-pentadione, 2-methyl-3-hexanone, and the majority of the esters (Table S3, Supplementary Materials).

### 3.3. Gut Permeability and Bowel Wall Analysis

**Figure 6:**
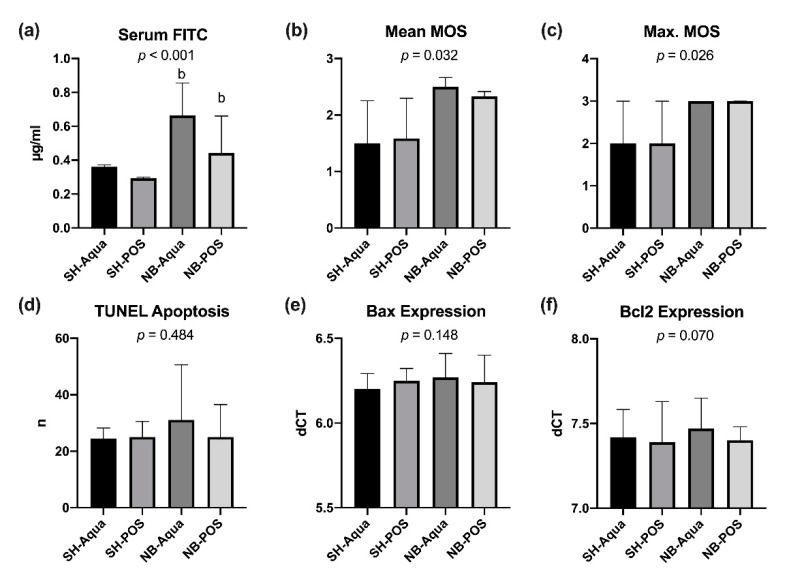
Analysis of bowel wall permeability; (**a**) serum FITC levels, (**b**) mean and (**c**) maximal Marsh–Oberhuber score (MOS), (**d**) TUNEL apoptosis, (**e**) Bax and (**f**) Bcl2 expression in the ileum. NB—neuroblastoma; SH—sham; POS—prebiotic oligosaccharide; b—*p* < 0.05 vs. SH-POS.

Tumor-bearing animals exhibited a higher permeability for FITC-dextran 4kD than animals of the sham groups (*p* < 0.001, Figure 6a). In a pairwise comparison, NB-Aqua and NB-POS showed significantly higher serum levels of FITC-dextran compared to SH-POS but not to SH-Aqua (Figure 6a). Prebiotic supplementation tended to reduce FITC permeability in tumor animals (NB-POS), but the difference did not reach statistical significance. Serum FITC levels showed a weak but significant positive correlation with IL-6 and o-xylene and a negative correlation with TGF-β1 and TGF-β2 levels but not with the remaining fecal VOCs (Table S4, Supplementary Materials).

In search of possible underlying reasons, bowel wall inflammation, mucin barrier, bowel wall apoptosis, and tight junction protein expression were evaluated in the ileum samples. The histological scoring of the bowel wall assessed by the Marsh–Oberhuber scores as a sign for bowel wall inflammation was significantly increased in tumor animals (NB-Aqua and NB-POS) compared to the control groups (Figure 6b,c).

The PCR for *mucins 2* and *3* as major components of small intestinal mucus revealed no significant group differences (data not shown). Apoptosis rates of IECs in the crypts were not significantly different between the groups (Figure 6d). The PCR for apoptosis markers revealed no statistically significant differences of the expression of pro-apoptotic *Bax* and *Bcl2* (Figure 6e,f). There were also no statistically significant alterations of the other apoptosis markers examined (data not shown).

Regarding the tight junctions, we could not observe any statistically significant differences for markers of the pore (*claudin 2, claudin 4, tight junction protein 1* and *occludin*) or leak (*myosin light chain kinase*) pathways (data not shown).

As none of the markers tested could explain the significant increase of FITC-dextran by itself, a multiple linear regression analysis predicting serum FITC levels from *MUC2, MUC3, Bad, Bax, lamin B, Bak, Bcl2, caspase 3, claudin 2, 4, MLCK, occludin, TJP-1*, apoptosis rate in TUNEL stains, and maximum Marsh–Oberhuber score was conducted. *TJP-1* was excluded from the analysis because of multicollinearity. The examined variables statistically significantly predicted serum FITC levels (F(14, 19) = 3.856; *p* = 0.004; R^2^ = 0.740). *Bax* and the maximum Marsh–Oberhuber score added significantly to the prediction (*p* < 0.05). 

Moreover, a multiple regression was run to predict FITC from the bacteria, which were relevant in the LEfSe analysis (*Muribaculum*, *Alloprevotella*, *Allistipes*, *Lactobacillus*, *Candidatus*, *AD3011*, *Roseburia*, *Tyzzerella 3*, *Eubacterium*, *Anaerotruncus*, *Butyricoccus*, *UCG13*, *UBA1819*, and *Erysipelatoclostridium*). These bacteria were able to significantly predict FITC, *F*(14, 23) = 2.225, *p* = 0.043, *R^2^* = 0.575. *Anaerotruncus* added significantly to the prediction, *p* < 0.05. 

## 4. Discussion

We demonstrated in a murine neuroblastoma model that prebiotic supplementation with OMNi-LOGiC^®^ FIBRE was associated with compositional changes in the fecal microbiome. As a key finding, prebiotic treatment led to an increase of Clostridial Family XIII AD3011 and a decrease of *Muribaculum* in tumor animals. Clinically, this may be associated with increased short-chain fatty acid (SCFA) production and decreased bowel wall inflammation exerting potential beneficial effects for oncological patients. Both the tumor and prebiotic treatment led to distinct alterations of the fecal VOC profile demonstrating associations between microbiome, VOCs, and host metabolism. 

OMNi-LOGiC^®^ FIBRE is a prebiotic consisting of dextrin (resistant starch type 4 with α-1,2- and α-1,3-linked dextrose) and partially hydrolyzed guar gum (β-1,4-linked mannose with side chains of α-1,6-linked galactose). In previous studies, dextrin increased the amount of cecal SCFAs and inhibited tumor growth in cell cultures and tumor development in mouse xenografts of HCT116 cell lines [29]. Guar gum lead to an increase of cecal SCFA, a reduction of pro-inflammatory cytokines, and an improved gut barrier in models of experimental colitis [30,31]. These beneficial effects served as the underlying reasons to choose OMNi-LOGiC^®^ FIBRE as a combination of these two fibers for the experiments performed in the present study.

Tumor-associated cachexia is defined as a catabolic state characterized by muscle and adipose tissue wasting [1]. Although the full picture of TAC is rarely seen in children, about half of the children with a malignant tumor suffer from malnutrition [32]. In particular, patients with stage III and IV neuroblastoma (NB)—the most common solid extracranial tumor in childhood—are at a high risk of undernourishment [33,34]. The murine model of NB-associated cachexia has already been established previously by the authors to gain a deeper insight into the mechanism of TAC in this disease [5,11]. 

Dextrin has been reported to induce mitochondrial production of reactive oxygen species and Bax-dependent cleavage of caspases 3 and 9, leading to decreased tumor cell growth in HCT116 (human colorectal carcinoma) cell cultures and to reduced tumor development in HCT116 mouse xenografts [29]. In contrast, our NB-bearing animals did not react to this substance regarding tumor take rate, tumor growth, or tumor weight. Different responses of MHH-NB11 and HCT116 cell lines could be hypothesized as a possible underlying reason. Similar to our previous reports, end-stage NB caused TAC with depletion of adipose tissue in tumor-bearing animals in this investigation [5,11]. In their experiments with inulin and pectin in a BAF3 leukemia mouse model, Bindels et al. reported decreased TAC-associated metabolic alterations with reduced loss of fat mass and decreased anorexia following treatment with pectin. Inulin but not pectin caused reduced cancer cell invasion to the liver. However, neither pectin nor inulin influenced tissue inflammation associated with TAC [9]. Similar to their results, OMNi-LOGiC FIBRE^®^ did not ameliorate the pro-inflammatory state associated with TAC in our model. In contrast to the report of Bindels and coworkers, we encountered neither reduced adipose tissue wasting nor improved anorexia following prebiotic treatment. Their results demonstrate that different prebiotics seem to exert different metabolic effects. This could also be an explanation for the discrepant findings of our investigation. 

Alterations of the gastrointestinal microbiome have been shown in a variety of oncologic diseases. Following prebiotic treatment, mice in our study demonstrated increased abundances of *Faecalibacterium UBA1819* and decreased abundances of *Anaerotruncus*, *Tyzzerella 3*, *Lactobacillus*, *Butyricoccus*, *Candidatus arthromitus*, *Alloprevotella*, and *Erysipelatoclostridium*. Likewise, treatment with different prebiotics was associated with increased abundance of *Faecalibacterium* in humans [35,36,37]. However, while *Faecalibacterium prausnitzii* is the most commonly described strain in humans, we found increases of *Faecalibacterium UBA 1819*, which have not been reported in the literature so far. In general, *Faecalibacteria* are butyrate-producing bacteria increasing the content of this SCFA in the large bowel [37]. 

Increased abundances of *Tyzzerella 3* have been described in patients with gestational diabetes and patients with increased lifetime risks of cardiovascular diseases as well as in rats with a high sugar diet [38,39,40]. Therefore, a decrease of *Tyzzerella 3* could be considered as beneficial. In their investigation of the effect of different high-fiber diets in rats with experimental bowel inflammation, Svolos et al. described increases of *Tyzzerella 3*, *Erysipelatoclostridium*, and *Anaerotruncus* (among others) and a decrease of *Lactobacillus* and *Candidatus*. While their findings for *Lactobacillus* and *Candidatus* are in line with our results, their data for the other listed bacteria such as *Tyzzerella 3* are contradictory [41]. A study by Cheng et al. performed in a murine model reported that the alterations of the intestinal microbiome depend on the type of fiber used [42]. While a combination of galacto-oligosaccharides and inulin resulted in an increase of Bacteroidetes and a decrease of *Alloprevotella*, the results were oppositional for a combination of poly-dextrose and dietary fiber from bran. Consequently, the choice of the optimal prebiotic fiber in oncological patients warrants further research. 

Mice with NB-associated TAC in our study had decreased abundances of *Erysipelatoclostridium*, *Eubacterium*, and *Roseburia* as well as increases of *Muribaculum*, *Faecalibacterium UBA1819*, and *Ruminococcaceae UCG013*. The reduction of butyrate-producing *Eubacterium* and *Roseburia* confirms reports demonstrating reduced relative abundances of these bacteria in patients with prostate or colorectal cancer [43,44]. The findings concerning *Ruminococcus* contradict reports of decreased abundances in patients with non-small cell lung or colorectal cancer [45,46]. Studies concerning *Muribaculum* are restricted to a murine model reporting its decreased relative abundance in SHIP-deficient mice with inflammatory bowel disease [47]. Thus, *Muribaculum* (among others) may be linked to inflammatory processes localized in the intestinal wall.

In our model, the administration of OMNi-LOGiC FIBRE^®^ (NB-POS animals) led to an increase of Clostridial Family XIII AD3011 and a decrease of *Muribaculum* compared to tumor animals receiving water only (NB-Aqua). An increase of Clostridial Family XIII as butyrate producers under a prebiotic diet has also been reported in humans with Crohn’s disease and rats with experimental bowel inflammation [41] and may have beneficial effects by stabilizing the intestinal barrier function. Besides increases of Clostridial Family XIII, we demonstrated a decrease of *Muribaculum* under prebiotic treatment. This may be associated with a reduction of inflammatory processes in the bowel wall. Histological examinations of the ileum samples, however, did not yield any significant effects of POS supplementation regarding tissue inflammation scores in this investigation. Overall, data in the literature in combination with our findings underline the complex interaction between tumor, inflammation, microbiome, and bacterial metabolism. 

Volatile organic compounds in the headspace of fecal samples are generated during metabolic processes within the gut and are influenced by intestinal epithelium, the microbiome, and diet [48]. Typically for fecal samples, esters dominated the VOC profile in our measurements. The tumor itself seemed to influence these volatile ester compounds because decreased levels in animals with TAC were found. In contrast, increased fecal levels of volatile esters have been previously shown in patients with inflammatory bowel syndrome (IBD) and non-alcoholic fatty liver disease [49,50,51]. Isopropylpropionate, propionic acid pentyl ester, and butanoic acid propyl ester, however, seemed to respond to both prebiotic treatment and TAC. The ester 1-methoxy-2-propylacetate was increased in animals supplemented with OMNi-LOGiC FIBRE^®^ (SH-POS) compared to mice receiving water. However, little is known about the biochemical pathways associated with the production of volatile esters in the human gut [50]. Similar to Raman and coworkers, we are not aware of any unique metabolic pathways linking the microbial changes observed to the production of fecal ester compounds [50]. Taken together, the reason for the discrepancies with low ester levels in TAC and high levels in non-alcoholic fatty liver disease and IBD remain unclear and are a subject for future investigations. The possibility to use esters as metabolic biomarkers during oncological therapy remains to be elucidated.

Ketones were the second most predominant group affected by NB-associated TAC with significantly lower levels of acetone, methylvinylketone, 3-pentanone, and 2-methyl-3-hexanone. Acetone is derived from acetacetate by acetyl-CoA originating either from lipolysis or glycolysis [52]. In this regard, acetone levels in exhaled breath have been inversely correlated to serum dextrose levels [52,53]. Increased urine levels of ketones can be found during fasting and in diabetic patients due to increased ketogenesis. Despite the known link to glucose metabolism, these mechanisms cannot explain decreased ketone levels in mice with TAC. However, Hueppe et al. demonstrated decreased levels of acetone and 3-pentanone—a metabolite of branched amino acid degradation—in an ischemia and reperfusion shock model [54]. They hypothesize a decreased enzyme activity during shock as the likely reason for their findings. Our experiments show a significant correlation between total WAT and fecal volatile ketone levels. Animals with normal WAT had higher ketone levels than those with a depletion of adipose tissue, possibly linking TAC (characterized by depletion of WAT) with reduced ketone levels. 

Ethanol in fecal samples is a result of bacterial fermentation [55]. We demonstrated a weak, but significant correlation between ethanol levels and the relative abundance of *Roseburia* and *Eubacterium*, which are known producers of ethanol (summarized in [56]) (Table S1, Supplementary Materials). Isopropanol originates among others from the reduction of acetone by hepatic alcohol dehydrogenase [52]. Similar to fecal volatile ketones, a reduced enzyme activity during TAC could be hypothesized as the reason for reduced isopropanol levels in tumor-bearing mice. 

Finally, inflammation and cancer-related stress can lead to increased endogenous production of alkenes and methylated alkanes as markers for lipid peroxidation [57]. This association can be a possible reason for the increased levels of octane encountered in mice with TAC. 

Similar to previous investigations by our group, mice with TAC exhibited significantly increased gut permeability for FITC-dextran linked to inflammatory cytokine levels [5]. In severe illness, various factors can lead to a disruption of the intestinal barrier function, for example, breakdown of the mucus layer, increased para-cellular permeability via the pore or leak pathway, loss of enterocyte integrity, and decreased intestinal immunity [58,59]. Therefore, we investigated key components of these pathways but could only demonstrate increased inflammation scores in the ileum samples of mice with TAC. Hence, we conducted a linear multiple regression analysis and found a significant link between FITC-dextran permeability and mucin expression (MUC2, MUC3), leak pathway (MLCK), pore pathway (claudin 2, 4, occludin, tight junction protein 1), apoptosis (Bad, Bax, lamin B, Bak, Bcl2, caspase 3, and apoptosis rate in TUNEL stains), and bowel wall inflammation (maximum Marsh–Oberhuber score). All of these variables statistically significantly predicted serum FITC levels. Bax and the maximum Marsh–Oberhuber score added significantly to the prediction. These results suggest that TAC-associated bowel wall hyperpermeability cannot be linked to a single parameter but seems to be triggered by a multitude of different factors.

Limitations of the present study include the fact that we focused on OMNi-LOGiC^®^ FIBRE as a combination of dextrin and partially hydrolyzed guar gum. Both fibers could also be tested individually in our model. However, this was not the focus of the present investigation and remains a future approach. Moreover, our examination of the bowel wall—especially regarding the mechanism for increased serum FITC levels in TAC—focused only on segments of the ileum. We decided to focus on the ileum similar to our previous investigation [5]. In this context, alterations of other intestinal regions could possibly have been missed. In our setting, it was impossible to examine the whole length of the intestine for all markers tested in this experiment. Therefore, future investigations should focus on the examination of the role of the other bowel segments. Additionally, results of VOC analysis are presented as peak areas of relevant mass traces rather than on concentration values, as not all identified VOC markers were available as pure reference substances. As the concentration of each VOC is directly related to the peak area (at a given retention time and mass trace), those values straightforwardly reflect concentration changes in the selected marker substances in the different groups.

In conclusion, we demonstrated that NB-induced TAC caused increased bowel permeability coupled with changes in the composition of the fecal microbiome and VOC profile. There were several links between metabolism, microbiome, and VOC profile circumstantiating the close relation between intestinal bacteria and their metabolism with metabolism and inflammation of the host. Prebiotic supplementation with OMNi-LOGiC^®^ FIBRE led to potentially positive modifications of the fecal microbiome and alterations of the VOC profile.

## Supplementary Materials

The following are available online at https://www.mdpi.com/2072-6643/12/7/2029/s1, Figure S1: Pairwise comparison of markers for beta diversity. (a) Effect of POS, (b) tumor effect and (c) effect of OMNi-LOGiC^®^ FIBRE in tumor mice, Table S1: Correlation analysis between microbiota and fecal ethanol levels, Table S2: Correlation analysis between fecal VOCs, liver weight, and muscle weight, Table S3: Correlation between fecal VOC levels, food intake, weight gain, and total WAT, Table S4: Correlation between fecal VOCs, inflammatory cytokines, and FITC permeability.
