# Supplementary figures and images for: The Effects of Prebiotic Supplementation with OMNi-LOGiC® FIBRE on Fecal Microbiome, Fecal Volatile Organic Compounds, and Gut Permeability in Murine Neuroblastoma-Induced Tumor-Associated Cachexia
Source: Nutrients. 2020 Jul 8;12(7):2029. doi: 10.3390/nu12072029 (PMC7400931; doi:10.3390/nu12072029)

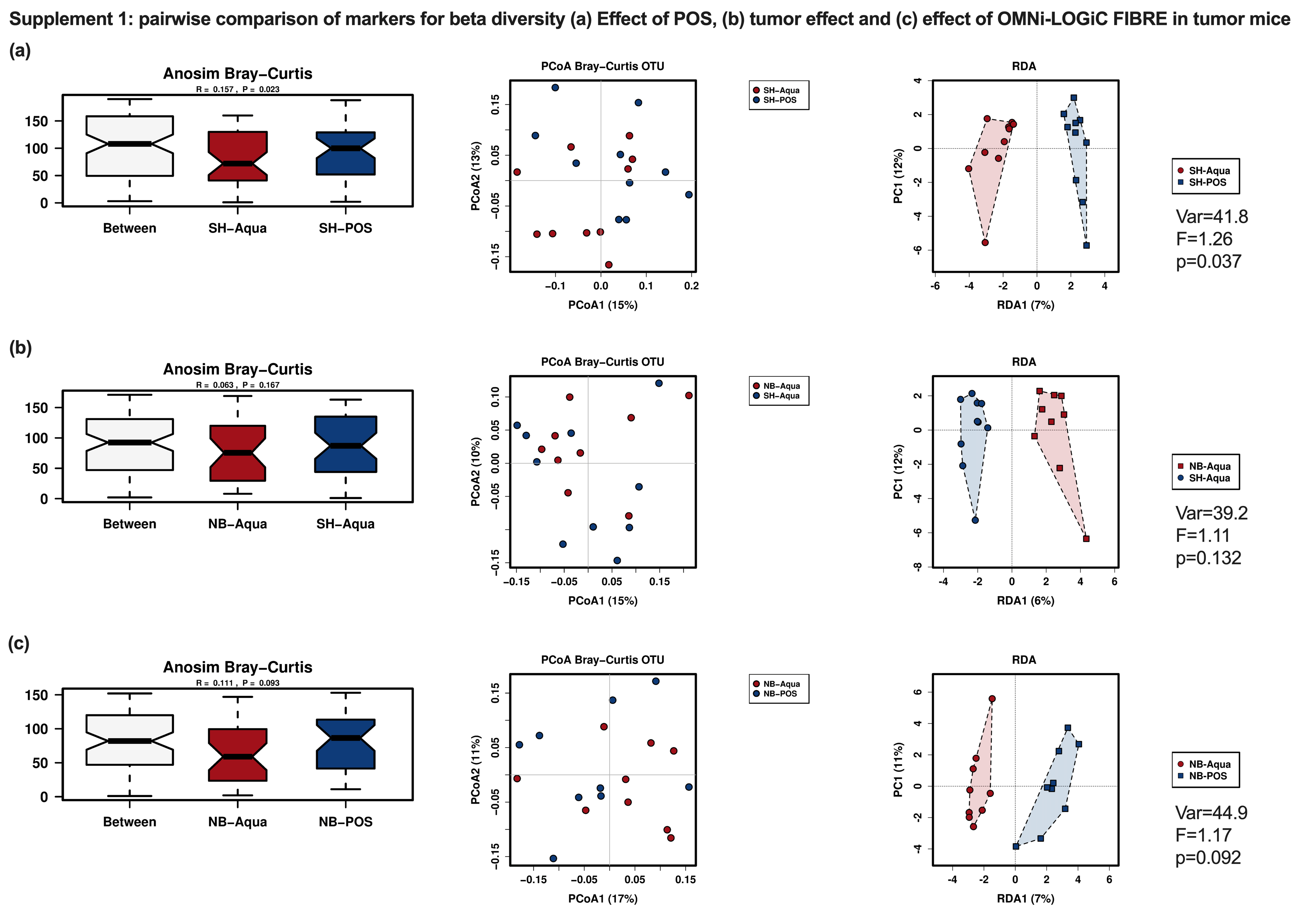

Supplement: Supplementary file 1 [file nutrients-12-02029-s001.zip › Suppl Files/Supplementary Figure S1.tiff]
